# Muscle oxygen saturation stratification by photoacoustic imaging in diabetic sarcopenia: Association with disease status

**DOI:** 10.1016/j.pacs.2025.100771

**Published:** 2025-09-27

**Authors:** Linxin Yang, Han Wu, Jing Chen, Wei Wang, Zhenxiu Zhang, Jiaping Feng, Wanbing Qiu, Fajin Dong, Ning Lin, Fengyi Yuan

**Affiliations:** a Ultrasound Department, Shenzhen Peoples Hospital (The Second Clinical Medical College, Jinan University, The First Affiliated Hospital, Southern University of Science and Technology), Shenzhen, Guangdong 518020, China; b Department of Endocrinology, Shenzhen Peoples Hospital (The Second Clinical Medical College, Jinan University, The First Affiliated Hospital, Southern University of Science and Technology), Shenzhen, Guangdong 518020, China; c Guangdong Provincial Clinical Research Center for Geriatrics, Shenzhen Clinical Research Center for Geriatrics, The Second Clinical Medical College, Jinan University (Shenzhen People’s Hospital), Shenzhen, Guangdong 518020, China; d Shengli Clinical Medical College of Fujian Medical University, Fujian Medical University, Fuzhou, Fujian, China; e Department of Ultrasound, Fuzhou University Affiliated Provincial Hospital, Fujian Provincial Hospital, Fuzhou, Fujian, China

**Keywords:** Sarcopenia, Photoacoustic Imaging, Muscle Oxygenation, Type 2 Diabetes

## Abstract

Sarcopenia, commonly observed in diabetes, is characterized by reduced muscle mass and function. However, the relationship between muscle oxygen saturation (SO₂) and sarcopenia remains unclear. Photoacoustic imaging (PAI) offers a promising method for assessment. This study aimed to evaluate SO₂ distribution in diabetic sarcopenic patients using PAI and explore associations with clinical parameters. Type 2 diabetes patients (≥ 10 years) underwent PAI of the gastrocnemius and vastus lateralis muscles. Sarcopenia was diagnosed by AWGS 2019/2020 criteria. SO₂ values stratified patients into hypoxia, intermediate, and hyperoxia groups. The study included 64 sarcopenic and 115 non-sarcopenic patients. Mean SO₂ was significantly lower in sarcopenia (63.28 % vs. 66.26 %, P < 0.001). PAI-measured SO₂ was an independent protective factor (β = −0.10, P = 0.001). In conclusion, PAI-assessed SO₂ is associated with sarcopenia and may serve as an early screening biomarker.

## 1 Introduction

Sarcopenia is a progressive and generalized skeletal muscle disorder defined by the accelerated loss of muscle mass and function [1], [2]. Individuals affected by sarcopenia are predisposed to functional decline, frailty, falls, and increased mortality, posing serious challenges to global public health, particularly in aging societies [1]. Moreover, sarcopenia is no longer considered an exclusive consequence of aging, but is also highly prevalent among patients with chronic conditions, especially type 2 diabetes mellitus (T2DM) [3]. The coexistence of T2DM and sarcopenia has been associated with compounded risks of disability, hospitalization, and poor metabolic control, underlining the urgent need for reliable diagnostic and monitoring tools in this population [4]. Based on accumulating evidence, skeletal muscle microcirculation impairment and tissue hypoxia — resulting from insulin resistance [5], chronic hyperglycemia, and diabetic microangiopathy [6] — play pivotal roles in the pathophysiology of sarcopenia in patients with T2DM [7]. These factors contribute to reduced oxygen delivery to muscle tissues, promoting muscle atrophy and functional decline.

However, direct, non-invasive assessment of muscle oxygenation remains challenging in routine clinical practice. Current diagnostic modalities for sarcopenia, such as bioelectrical impedance analysis (BIA) and dual-energy X-ray absorptiometry (DXA) [8], [9], primarily assess muscle quantity but offer limited information on tissue metabolic status and oxygenation. In recent years, ultrasound-based modalities have shown promise for muscle evaluation due to their convenience, non-invasiveness, and ability to assess both structural and mechanical properties of muscle tissue [10], [11]. B-mode ultrasound and shear wave elastography (SWE) have been successfully applied to quantify muscle thickness and stiffness [12], [13], which indirectly reflect muscle quality. Nevertheless, these techniques do not directly capture muscle tissue oxygenation [11] status, which may provide more comprehensive insight into muscle health.

PAI has emerged as a novel hybrid imaging modality that combines the high optical contrast of optical imaging with the deep tissue penetration and spatial resolution of ultrasound [14]. By detecting acoustic waves generated through the photoacoustic effect following the absorption of pulsed laser light, PAI enables quantitative visualization of endogenous chromophores such as oxygenated and deoxygenated hemoglobin [15]. This allows for non-invasive, in vivo measurement of tissue SO_2_ without the need for exogenous contrast agents [16], [17]. Furthermore, PAI offers real-time imaging capabilities and excellent spatial resolution at clinically relevant depths, which makes it highly attractive for biomedical research and clinical translation [18].

In recent years, PAI has been increasingly integrated into multimodal ultrasound systems, expanding its application scope in the field of sonography. Notably, PAI has been successfully applied in oncology to assess tumor hypoxia, in rheumatology to evaluate synovial inflammation and vascularization in rheumatoid arthritis [19], [20], in breast imaging to characterize malignant lesions [21], and in vascular medicine to visualize microvascular networks and arterial plaques [22]. PAI and multispectral optoacoustic tomography (MSOT) have emerged as powerful tools for non-invasive assessment of muscle hemodynamics and oxygenation. Previous studies have successfully employed this technology in healthy volunteers under physiological stressors [23] and in neuromuscular and vascular diseases such as Duchenne muscular dystrophy and peripheral artery disease [24], [25], [26]. Building upon this foundation, our study aims to explore the novel application of PAI for evaluating muscle oxygenation impairments in the context of diabetic sarcopenia, a common yet underdiagnosed complication of diabetes characterized by progressive loss of muscle mass and function.

Despite its potential, few studies have explored the application of PAI in the context of sarcopenia, particularly among high-risk populations such as long-term T2DM patients. Therefore, the present study aims to fill this knowledge gap by utilizing PAI to assess muscle oxygen saturation in the gastrocnemius and vastus lateralis muscles in T2DM patients with and without sarcopenia. Sarcopenia was diagnosed based on AWGS 2019/2020 criteria [27], incorporating muscle mass, grip strength, and physical performance. Furthermore, unsupervised clustering analysis was applied to stratify sarcopenic patients into oxygenation phenotypes (hypoxia, intermediate, and hyperoxia), and the relationships between oxygen saturation levels, functional indicators, and sarcopenia risk were systematically investigated. This study seeks to determine whether muscle oxygenation measured by PAI could serve as a valuable imaging biomarker for sarcopenia screening and risk stratification in T2DM patients.

## 2 Materials and methods

This prospective observational cohort study was conducted at Shenzhen People's Hospital in collaboration with Mindray Biomedical Electronics, who assisted in the development of the PAI system. In accordance with the ethical guidelines of the Declaration of Helsinki, written informed consent was obtained from all participants prior to enrollment. The imaging data were collected and processed independently by non-employee investigators who had full control over the research data and analysis.

### 2.1 Participants

**Fig. 1:**
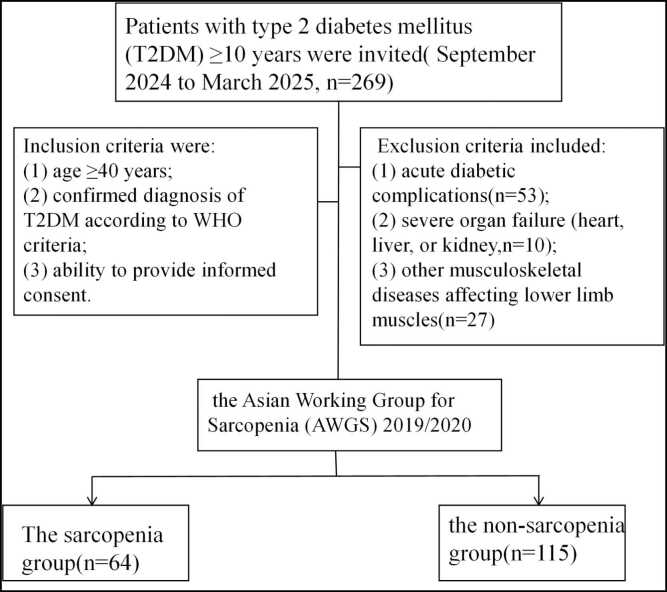
Flowchart of patient enrollment and study design. This flowchart details the step-by-step process of patient selection, including the initial screening of participants, the exclusion criteria applied, and the final number of patients included in the sarcopenia and non-sarcopenia groups for the study.

This is a cross-sectional observational study from September 2024 to March 2025 at Shenzhen People's Hospital. Patients with T2DM) ≥ 10 years were invited. Inclusion criteria were: (1) age ≥ 40 years; (2) confirmed diagnosis of T2DM according to WHO criteria; and (3) ability to provide informed consent. Exclusion criteria included: (1) acute diabetic complications (n = 53); (2) severe organ failure (heart, liver, or kidney, n = 10); and (3) other musculoskeletal diseases affecting lower limb muscles (n = 27) (Fig. 1).

Participants were classified into sarcopenia and non-sarcopenia groups based on the diagnostic criteria of AWGS 2019/2020. Sarcopenia was defined by the presence of low muscle mass (assessed by bioelectrical impedance analysis, BIA, low muscle strength (grip strength), and/or poor physical performance (6-meter walking speed). The non-sarcopenia group included participants who did not meet these criteria.

### 2.2 PAI for Muscle Oxygenation

PAI was performed using a commercially available ultrasound system (Mindray Bio-Medical Electronics, Shenzhen, China) equipped with a tunable optical parametric oscillator (OPO) laser (InnoLas Laser, Krailling, Germany) and a handheld linear PA/US L9–3 probe. Dual-wavelength PAI was conducted at 750 nm and 830 nm to quantify tissue SO_2_. The acquired photoacoustic images were presented as pseudo-color maps superimposed on grayscale ultrasound images. Red and blue colors corresponded to high and low values, respectively, visually indicating oxygenation status within the muscle tissue. The SO_2_ values (hereafter referred to as SO₂) were automatically calculated by an integrated algorithm based on the photoacoustic signals.

To reduce random noise, each acquisition was recorded as a 5-second video after image stabilization. A rectangular ROI (approximately 2 × 4 cm) was placed at the thickest part of the vastus lateralis and gastrocnemius muscles, avoiding large vessels. For each site, 3 frames were extracted and averaged to obtain the final SO₂ value. Photoacoustic images were reconstructed using the proprietary algorithm integrated into the commercial system (Mindray Bio-Medical Electronics), with standard band-pass filtering automatically applied to enhance the signal-to-noise ratio. For detailed information on the formulas and processing steps for SO2 measurements, see Appendix E1.

### 2.3 Image Assessment and SO₂ Subgroup Classification

Two senior ultrasound physicians, Dr. Dong (15 years of experience in musculoskeletal ultrasound) and Dr. Chen (10 years of experience), performed multimodal photoacoustic/ultrasound (PA/US) imaging of the vastus lateralis and gastrocnemius muscles. They were aware of the participants' names and ages but were blinded to their medical history, laboratory results, and sarcopenia status. Standard B-mode ultrasound was first performed to assess muscle morphology, followed by PAI to evaluate muscle SO_2_ at four predefined anatomical sites for each patient, including the transverse and longitudinal planes of the vastus lateralis and gastrocnemius muscles. The overall muscle oxygenation was expressed as SO_2_, representing the mean SO₂ across the four sites. For each site, the ultrasound physicians performed three measurements, and the average SO₂ was calculated. To investigate oxygenation patterns, unsupervised K-means clustering analysis was performed on SO_2_ values in sarcopenic patients. According to the elbow method and clustering performance indices, the optimal number of clusters was determined to be three. Two additional senior ultrasound physicians, Dr. Feng (15 years of experience in musculoskeletal ultrasound) and Dr. Zhang (10 years of experience), who were blinded to all clinical information, independently reviewed the pseudo-color images and confirmed the classification results based on SO_2_ values and color patterns. Using this combined quantitative and qualitative approach, the oxygenation status of skeletal muscle in sarcopenic patients was categorized into three phenotypes: hypoxia, intermediate oxygenation, and hyperoxia.

### 2.4 Clinical Assessment

Clinical evaluation of sarcopenia was performed by two senior endocrinologists (Dr. Yuan and Dr. Wu), each with more than 20 years of experience in endocrinology. Comprehensive assessments were conducted according to the diagnostic criteria of AWGS 2019/2020. The evaluation included measurement of muscle mass, muscle strength, and physical performance. Muscle mass was assessed using BIA, with skeletal muscle mass index (SMI) calculated accordingly. Muscle strength was determined through handgrip strength testing using a handheld dynamometer, with the better value of two trials from the dominant hand recorded. Physical performance was assessed by measuring the usual gait speed over a 6-meter walking distance. In addition, other clinical indicators such as body mass index (BMI) and calf circumference were measured as supplementary parameters for muscle quantity and nutritional status. Based on the integrated results of these assessments, participants were classified as having or not having sarcopenia according to AWGS criteria. All clinical assessments were conducted in a standardized manner by the endocrinologists, who were blinded to the PAI results.

### 2.5 Statistical Analysis

All statistical analyses were performed using R software (version 4.2.2, R Foundation for Statistical Computing). Principal component analysis (PCA) was performed using the prcomp function in R, with centering and scaling applied, to visualize the separation of the K-means clusters in a two-dimensional space. PCA was used to visualize the separation of the K-means clusters in a two-dimensional space, providing a clear depiction of muscle oxygenation subgroups (low, intermediate, and high oxygenation). Normally distributed variables are presented as means ± standard deviations (SD) and were compared using the Student t test for two-group comparisons or one-way analysis of variance (ANOVA) for multiple group comparisons. When ANOVA indicated significant differences among the three oxygenation subgroups (low, intermediate, and high oxygenation), post hoc pairwise comparisons were conducted using the Student t test. Non-normally distributed continuous variables are expressed as medians and interquartile ranges (IQR) and were compared using the Mann–Whitney *U* test for two groups or Kruskal–Wallis H test for multiple group comparisons. Dunn’s post hoc test with Bonferroni correction was performed when the Kruskal–Wallis test showed significant differences across groups. Categorical variables were analyzed using the chi-square test or Fisher’s exact test, as appropriate. Bonferroni correction was applied to adjust for multiple comparisons, and a two-sided P-value of < 0.05 was considered statistically significant. Additionally, receiver operating characteristic (ROC) curve analysis was performed to evaluate the predictive performance of SO₂ for sarcopenia diagnosis.

## 3 Result

### 3.1 Baseline Characteristics of Participants

A total of 179 participants were included in the analysis, comprising 64 non-sarcopenic and 115 sarcopenic patients. The baseline characteristics of the two groups are summarized in Table 1. Similarly, sarcopenic patients had significantly lower values in several anthropometric and functional indicators, including calf circumference, BMI, muscle mass, body weight, hip circumference, and grip strength (all P < 0.001). Furthermore, they exhibited significantly higher levels of UACR (P = 0.003) and a lower estimated glomerular filtration rate (eGFR) (P = 0.004), suggesting poorer renal function. Older age (P = 0.02) and lower hemoglobin (HGB) levels (P = 0.02) were also more frequently observed in the sarcopenic group. Additionally, the prevalence of hypertension (P = 0.03), anemia, and diabetes-related complications was higher among sarcopenic participants. Taken together, these results demonstrate that sarcopenia in patients with long-standing T2DM was associated with older age, reduced muscle quantity and strength, poorer physical performance, and impaired metabolic and renal profiles.

**Table 1:** Sarcopenia patients and clinical baseline table of patients with sarcopenia.

Variables	Total (n = 179)	Non-Sarcopenia (n = 64)	Sarcopenia (n = 115)	statistic	p
Gender, n (%)				6.85	0.009
Male	110 (61.45)	48 (75)	62 (53.91)		
Female	69 (38.55)	16 (25)	53 (46.09)		
Height, Median (Q1,Q3)	160 (153, 165)	161 (157, 166.50)	159 (150.5, 164)	6.47	0.011
Weight, Median (Q1,Q3)	57.6 (50.95, 64.40)	65.95 (59.43, 72.90)	53.6 (48, 58.40)	62.91	< 0.001
BMI, Mean ± SD	22.85 ± 3.09	25.45 ± 2.52	21.40 ± 2.34	116.46	< 0.001
Age, Mean ± SD	65.63 ± 8.75	67.47 ± 7.66	64.60 ± 9.18	4.50	0.04
Location, n (%)				0.44	0.51
Left	80 (44.69)	26 (40.62)	54 (46.96)		
Right	99 (55.31)	38 (59.38)	61 (53.04)		
Waistline, Mean ± SD	87.68 ± 9.90	94.08 ± 7.80	84.13 ± 9.15	53.85	< 0.001
Calf, Median (Q1,Q3)	32 (31, 34)	35 (33, 37)	31.50 (30, 32)	73.06	< 0.001
Hips, Median (Q1,Q3)	92 (86.25, 96)	96 (92, 100)	89 (84.50, 92.50)	55.82	< 0.001
Mass, Median (Q1,Q3)	6.38 (5.56, 6.73)	6.9 (6.41, 7.54)	5.68 (5.36, 6.53)	64.11	< 0.001
Grip, Median (Q1,Q3)	22.4 (16.70, 29)	23.80 (19.98, 29.72)	20.70 (16.20, 27.80)	4.61	0.03
Smoke, n (%)				3.11	0.08
Yes	30 (16.76)	6 (9.38)	24 (20.87)		
No	149 (83.24)	58 (90.62)	91 (79.13)		
Speed, Mean ± SD	0.83 ± 0.22	0.86 ± 0.24	0.81 ± 0.21	1.82	0.18
DM, Median (Q1,Q3)	13 (5, 20)	13.5 (7.75, 20)	12 (4, 20)	2	0.16
Drink, n (%)				6.42	0.01
Yes	33 (18.44)	5 (7.81)	28 (24.35)		
No	146 (81.56)	59 (92.19)	87 (75.65)		
Hypertension, n (%)				4.93	0.03
Yes	85 (47.49)	38 (59.38)	47 (40.87)		
No	94 (52.51)	26 (40.62)	68 (59.13)		
CKD, n (%)				2.98	0.08
Yes	22 (12.29)	12 (18.75)	10 (8.70)		
No	157 (87.71)	52 (81.25)	105 (91.30)		
CAD, n (%)				0	1
Yes	25 (13.97)	9 (14.06)	16 (13.91)		
No	154 (86.03)	55 (85.94)	99 (86.09)		
DKD, n (%)				0.35	0.55
Yes	23 (12.85)	10 (15.62)	13 (11.30)		
No	156 (87.15)	54 (84.38)	102 (88.70)		
Hyperlipidemia, n (%)				Fisher	1
Yes	168 (93.85)	60 (93.75)	108 (93.91)		
No	11 (6.15)	4 (6.25)	7 (6.09)		
Retinopathy, n (%)				0.601	0.44
Yes	64 (35.75)	20 (31.25)	44 (38.26)		
No	115 (64.25)	44 (68.75)	71 (61.74)		
Blood, n (%)				Fisher	0.01
Yes	166 (92.74)	55 (85.94)	111 (96.52)		
No	13 (7.26)	9 (14.06)	4 (3.48)		
Neuropathy, n (%)				0.93	0.33
Yes	140 (78.21)	47 (73.44)	93 (80.87)		
No	39 (21.79)	17 (26.56)	22 (19.13)		
Bone density, n (%)				1.71	0.43
normal	42 (23.46)	15 (23.44)	27 (23.48)		
decrease	80 (44.69)	25 (39.06)	55 (47.83)		
Osteoporosis	57 (31.84)	24 (37.50)	33 (28.70)		
Creatinine, Median (Q1,Q3)	65 (54.50, 81)	73 (54.50, 92.5)	63 (54.50, 76.50)	3.37	0.07
eGFR, Median (Q1,Q3)	89.24 (73.98, 97.96)	81.72 (61.98, 95.69)	91.25 (80.08, 100.21)	8.23	0.004
Blood sugar, Median (Q1,Q3)	6.83 (5.50, 7.68)	6.94 (5.68, 7.57)	6.61 (5.42, 7.92)	0.06	0.80
TC, Median (Q1,Q3)	4.58 (3.69, 5.50)	4.46 (3.98, 5.20)	4.77 (3.58, 5.55)	0.36	0.55
HDL_C, Median (Q1,Q3)	1.17 (0.97, 1.35)	1.16 (0.94, 1.27)	1.19 (0.98, 1.37)	2.57	0.11
TG, Median (Q1,Q3)	1.18 (0.84, 1.81)	1.2 (0.89, 1.79)	1.18 (0.75, 1.84)	0.89	0.35
Uric acid, Median (Q1,Q3)	340 (283, 398)	345 (288.75, 434)	334 (282, 392)	0.63	0.43
Calcium, Mean ± SD	2.33 ± 0.12	2.35 ± 0.12	2.31 ± 0.11	2.76	0.10
Albumin, Mean ± SD	40.62 ± 4.1	40.32 ± 4.09	40.78 ± 4.12	0.52	0.47
Phosphorus, Median (Q1,Q3)	1.2 (1.09, 1.33)	1.24 (1.10, 1.40)	1.17 (1.08, 1.29)	6.15	0.01
Glycated hemoglobin, Median (Q1,Q3)	7.9 (6.70, 10.60)	8.35 (6.77, 11.43)	7.8 (6.70, 10.30)	0.49	0.49
C_peptide, Median (Q1,Q3)	1.67 (1.10, 2.50)	1.73 (1.05, 3)	1.67 (1.14, 2.42)	0.90	0.34
UACR, Median (Q1,Q3)	12.01 (5.58, 26.01)	16.74 (8.43, 64.76)	8.76 (5.21, 20.43)	8.90	0.003
25(OH)D, Median (Q1,Q3)	85.59 (59.43, 106.27)	86.94 (53.54, 118.42)	84.39 (59.95, 103.81)	0.25	0.62
HGB, Median (Q1,Q3)	132 (124, 139.50)	128 (120, 137)	134 (126.50, 140)	5.35	0.02
LDL_C, Mean ± SD	2.85 ± 0.91	2.8 ± 0.72	2.88 ± 1	0.31	0.58

T2DM: Type 2 diabetes mellitus; BMI: Body mass index; eGFR: Estimated glomerular filtration rate; UACR: Urinary albumin-to-creatinine ratio; HGB: Hemoglobin; Mass: Evaluation using; BIA: Bioelectrical Impedance Analysis；Grip: Grip strength; Speed: 6-meter walking speed; CKD: Chronic kidney disease; CAD: Coronary artery disease; DKD: Diabetic kidney disease; TC: Total cholesterol; TG: Triglyceride; HDL-C: High-density lipoprotein cholesterol; LDL-C: Low-density lipoprotein cholesterol; 25(OH)D: 25-hydroxyvitamin D; AWGS: Asian Working Group for Sarcopenia; PAI: Photoacoustic imaging

To explore the associations of baseline characteristics with the different muscle oxygenation phenotypes, we subsequently compared variables that showed statistically significant differences (P < 0.05) between the two groups across the three oxygenation subgroups. These variables included sex, height, age, calf circumference, BMI, muscle mass, weight, hip circumference, grip strength, UACR, eGFR, hemoglobin, and hypertension.

### 3.2 SO₂ Oxygenation Phenotypes Based on K-means Clustering

**Fig. 2:**
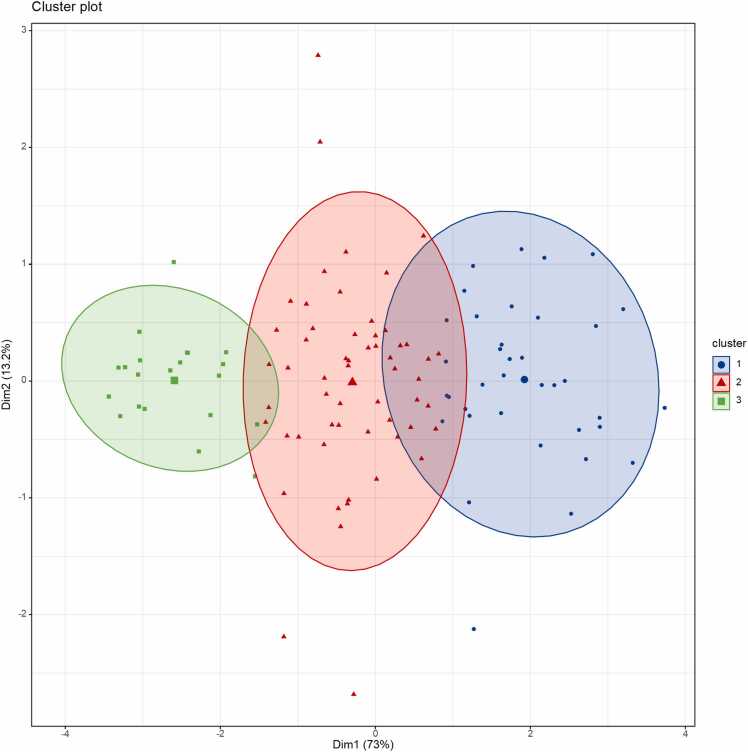
K-means clustering of skeletal muscle SO₂ in patients with sarcopenia. Principal component analysis (PCA)-based scatterplot demonstrating the K-means clustering results of sarcopenic patients based on average skeletal muscle SO₂ values measured by photoacoustic imaging. Patients were classified into three oxygenation phenotypes: low oxygenation (green dots), intermediate oxygenation (red dots), and high oxygenation (blue dots). Ellipses indicate the distribution range of each cluster. The separation of clusters reflects the heterogeneity of muscle oxygenation status among sarcopenic individuals.

K-means clustering analysis stratified sarcopenic patients into three distinct oxygenation phenotypes based on SO₂ values. The low oxygenation group (Class 1) exhibited SO₂ values ranging from 53.4 % to 59.6 %, with a mean of 56.2 % (n = 21). The intermediate oxygenation group (Class 2) showed SO₂ values between 59.9 % and 66.9 %, averaging 63.4 % (n = 57). In contrast, the high oxygenation group (Class 3) demonstrated the highest SO₂ values, ranging from 67.2 % to 76.2 %, with a mean of 70.4 % (n = 37). To provide an independent validation of the cluster separation, we projected the data onto a two-dimensional space using Principal Component Analysis (PCA) (Fig. 2). These two components together explained 86.38 % of the total variance. The three clusters were clearly separated, with green, red, and blue color-coded ellipses representing the low, intermediate, and high oxygenation groups, respectively. The spatial distribution of the clusters demonstrated that patients with low SO₂ values were positioned predominantly on the left side of the PCA plot (green group), whereas those with intermediate and high SO₂ levels occupied the middle (red group) and right (blue group) regions, respectively. These results indicate that sarcopenic patients exhibited considerable heterogeneity in muscle oxygenation status. Moreover, PAI combined with unsupervised clustering algorithms effectively stratified these patients into biologically and clinically relevant oxygenation phenotypes, which may reflect underlying pathophysiological differences in muscle microcirculation and tissue oxygen supply.

### 3.3 Comparison of SO₂ Values between Sarcopenic and Non-Sarcopenic Patients and among Sarcopenia Oxygenation Subtypes

In order to investigate the potential clinical implications of muscle oxygenation heterogeneity, clinical and functional parameters were compared among the three oxygenation phenotypes defined by K-means clustering (Table 2). Across the three oxygenation groups, a trend towards better clinical and functional profiles was observed with increasing SO₂ levels. Specifically, patients in the high-oxygenation group exhibited significantly higher BMI values (21.4 [19.8, 23.3]) compared to the intermediate-oxygenation group (22.9 [21.0, 23.7]) and the hypoxia group (20.5 [19.2, 22.9]), with pairwise comparison showing statistical significance (P = 0.02). Although calf circumference and muscle mass did not show significant differences across groups (P > 0.05), a tendency towards higher values was still noted in the high-oxygen group.

**Table 2:** Comparison of clinical and biochemical variables among the three classes.

paramter	hyperoxia group(n = 34)	intermediate group(n = 37)	hypoxia group(n = 44)	P Value	P Value*	P Value§	P Value†
Calf	31.50(30,33)	31.50(30,32)	31.50(30.00,32.10)	0.56	0.91	1	1
BMI	21.40(19.80,23.30)	22.90(21,23.70)	20.50(19.20,22.90)	0.02	0.59	0.54	0.02
Mass	6.20(5.50,6.60)	5.60(5.40,6.40)	5.70(5.30,6.50)	0.42	0.65	0.91	1
Weight	54.10(50.40,59.40)	54.0(50.90,60.10)	51.20(46.10,56)	0.13	1	0.25	0.28
Hips	89 (86.10,92.80)	92(85,93)	86.50(82,90.50)	0.12	1	0.26	0.21
Waistline	87.20(80,89.90)	86 (80,90)	83.50(77,86.20)	0.01	1	0.03	0.05
Grip	22.40(19.80,29.80)	17.10(15.70,29.70)	19.30(16.10,32.00)	0.07	0.06	0.90	0.48
Height	161(153,166)	156 (150,162)	158 (150,162)	0.41	0.72	0.73	1
Age	65.50(59, 74)	65 (58, 68)	63 (58, 67.80)	0.37	1	0.48	1
eGFR	91.80(75.90,101)	91.20(84.40,98.50)	89.40(79.80,98.70)	0.80	1	1	1
Phosphorus	1.17(1.03,1.28)	1.14(1.04,1.22)	1.20(1.14,1.33)	0.04	0.75	0.59	0.03
UACR	8.54(6.11,24.70)	15 (6.32,26)	6.18(3.32,14.60)	0.03	0.10	0.35	0.02
HGB	133 (126,140)	134 (126,140)	134 (126,139)	0.80	1	1	1
Drink	---	---	---	0.42	---	---	---
Blood	---	---	---	0.38	---	---	---
Gender	---	---	---	0.38	---	---	---
Hypertension	---	---	---	0.68	---	---	---

§Comparison of the hyperoxia and hypoxia oxygenation groups.

†Comparison of the intermediate and hypoxia oxygenation groups.

* Comparison of the hyperoxia and intermediate oxygenation groups.

**Fig. 3:**
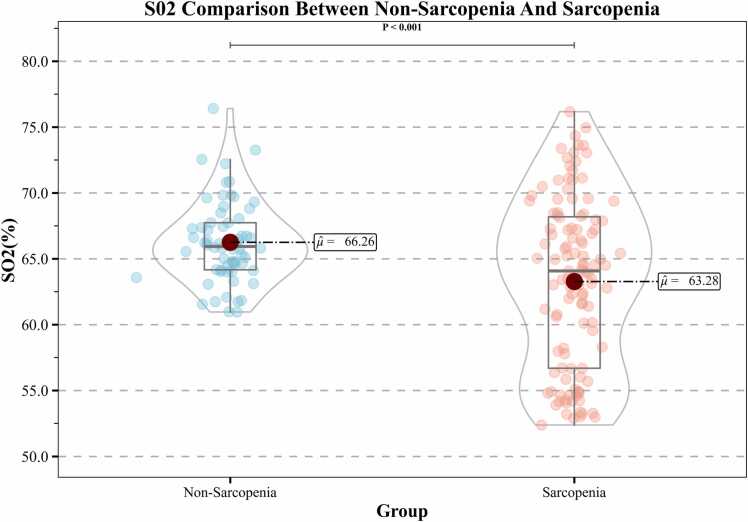
Comparison of SO₂ between non-sarcopenic and sarcopenic patients. Violin plots depicting the distribution of photoacoustic imaging-derived skeletal muscle oxygen saturation SO₂ values in non-sarcopenic and sarcopenic patients. The white dots represent the median values, the thick black bars indicate the interquartile range, and the thin black lines denote the range. Patients with sarcopenia demonstrated significantly lower SO₂ values compared with non-sarcopenic patients, suggesting that reduced muscle oxygenation is associated with sarcopenia.

Furthermore, when comparing patients with sarcopenia and those without sarcopenia, significant differences in muscle oxygenation were observed. The mean SO₂ was 63.28 % (range, 61 % – 76.20 %) in the sarcopenic group and 66.26 % (range, 52.40 % – 76.20 %) in the non-sarcopenic group. Although the difference in mean values appeared small, the distribution was more left-shifted in the sarcopenic group, suggesting a greater proportion of patients with reduced muscle oxygenation. The difference between the two groups reached statistical significance (P < 0.001), indicating that lower muscle oxygenation is associated with sarcopenia (Fig. 3).

### 3.4 Correlations of Muscle Oxygenation with Sarcopenia-Related Parameters

We found a nearly nonexistent correlation with total muscle mass (r = 0.08), indicating that SO_2_ is largely independent of this body composition indicator. Similarly, there was no significant correlation between SO_2_ and 6-meter walking speed (r = −0.02). These findings suggest that muscle oxygenation, as measured by our PA system, is not a direct correlate of general physical performance or body mass in this cohort.

**Fig. 4:**
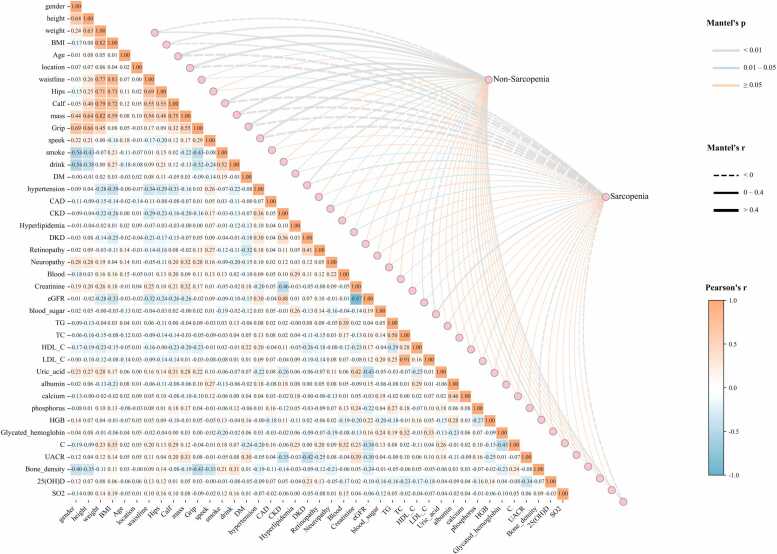
Correlation matrix of SO₂ and sarcopenia-related parameters. The figure presents the Pearson correlation coefficients between the average muscle SO_2_ and various clinical, functional, and anthropometric parameters. The color and size of the circles indicate the strength and direction of the correlation. Red circles represent a positive correlation, while blue circles indicate a negative correlation. The numbers within the circles are the correlation coefficients.

We have expanded our analysis to include a wide range of other patient characteristics, confirming that SO_2_ shows no strong correlation with anthropometric measurements like BMI (r = 0.19), waistline (r = 0.01), hips (r = 0.16), and calf circumference (r = 0.10) (Fig. 4).

### 3.5 Predictive Performance of PA-SO₂ for Diagnosing Sarcopenia

**Fig. 5:**
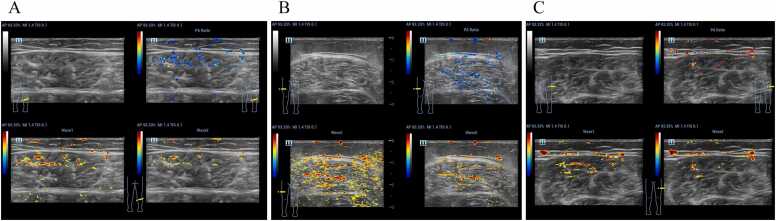
A:68-year-old male with severe sarcopenia. The PA/US images show predominantly blue pseudo-color signals with marked muscle thinning, increased echogenicity, and blurred fascial interfaces; B:62-year-old female with moderate sarcopenia. Mixed red and blue pseudo-color mapping is observed with mildly reduced muscle thickness and mildly heterogeneous echotexture; C:59-year-old female with mild or no sarcopenia. PA/US images show red-dominant pseudo-color signals with preserved muscle thickness, uniform echogenicity, and clearly defined structures.

To evaluate the diagnostic value of skeletal musclein identifying sarcopenia, a receiver operating characteristic (ROC) curve analysis was performed using SO₂ as the predictor variable. The results showed that SO₂ was significantly associated with sarcopenia diagnosis. The area under the ROC curve (AUC) was 0.63. Furthermore, logistic regression analysis demonstrated that SO₂ served as an independent protective factor against sarcopenia. Specifically, for every 1 % increase in SO₂, the risk of sarcopenia decreased by approximately 10 % (β = −0.10, P = 0.001). These findings suggest that reduced muscle oxygenation is independently associated with an increased risk of sarcopenia (Fig. 5).

## 4 Discussion

This study explored the clinical significance of muscle oxygenation assessed by PAI) in patients with sarcopenia complicated by T2DM. Through clustering analysis, we categorized patients into distinct oxygenation phenotypes and revealed meaningful associations with clinical and functional parameters. Our findings not only expand current knowledge of the pathophysiology of sarcopenia but also provide new insights into the potential application of PAI in muscle-related metabolic disorders.

In our protocol, the gastrocnemius and vastus lateralis muscles were selected for SO₂ measurement. These muscles play essential roles in locomotion and maintaining postural stability [28], [29], [30]. As primary weight-bearing and mobility-related muscles, they are particularly vulnerable to the deleterious effects of sarcopenia [31]. Furthermore, these large lower limb muscles are more susceptible to microvascular dysfunction and impaired perfusion in patients with T2DM [32], in whom chronic hyperglycemia and insulin resistance induce endothelial dysfunction, capillary rarefaction, and tissue hypoxia [33]. Therefore, targeting these muscles provided a representative and clinically meaningful assessment of muscle oxygenation status. When comparing muscle SO₂ between sarcopenic and non-sarcopenic patients, our results demonstrated that the sarcopenic group exhibited lower SO₂ levels. Although the mean SO₂ difference appeared modest, the sarcopenic population showed a broader distribution skewed toward lower values, suggesting that local muscle hypoxia may be more prevalent in sarcopenia. These results align with previous findings from inflammatory and metabolic conditions, where reduced tissue oxygenation is recognized as a driver of cellular dysfunction [34], [35]. Chronic hypoxia in skeletal muscle has been linked to mitochondrial impairment [36], reduced protein synthesis [37], and increased proteolysis — all of which contribute to muscle wasting and decreased strength [38]. To further investigate the heterogeneity of oxygenation among sarcopenic patients, we employed K-means clustering, which identified three oxygenation phenotypes: low, intermediate, and high. Furthermore, patients classified into the low-oxygenation group displayed poorer clinical and functional characteristics compared with those in the intermediate and high-oxygenation groups. Notably, BMI was significantly lower in the low-oxygenation group (P = 0.02), reflecting more advanced muscle loss and malnutrition [39]. Waist circumference was also reduced (P = 0.05), suggesting diminished nutritional reserves. Although grip strength did not reach statistical significance (P = 0.07), the observed downward trend supports the hypothesis that impaired oxygen delivery may compromise muscle function. Interestingly, UACR values were lower in the hypoxia group (P = 0.03), which may be explained by reduced muscle mass and lower creatinine production, although this finding warrants cautious interpretation. Collectively, these results highlight that reduced muscle oxygenation is associated with diminished muscle quantity and function, as well as altered systemic metabolic profiles. These observations support the hypothesis that impaired muscle oxygenation is not merely a consequence of sarcopenia but may also act as a contributing factor exacerbating muscle dysfunction [40]. Mechanistically, chronic muscle hypoxia can downregulate anabolic pathways, promote oxidative stress, and impair neuromuscular coupling, thereby perpetuating the cycle of muscle weakness and atrophy [41].

Moreover, our expanded correlation analysis further clarified the relationship between SO₂ and sarcopenia-related parameters. We found that SO₂ showed only weak correlations with total muscle mass (r = 0.08) and 6-meter walking speed (r = −0.02), suggesting that muscle oxygenation is not a direct surrogate of gross body composition or physical performance in this T2DM cohort. Similarly, SO₂ demonstrated no strong association with anthropometric parameters such as BMI, waistline, hip circumference, or calf circumference. These findings imply that PAI-derived SO₂ captures unique aspects of local microcirculation and oxygen delivery that are not adequately reflected by conventional measures of muscle mass or strength. Importantly, although no statistically significant correlations were observed, trends between SO₂ and biochemical indicators such as waistline, UACR, eGFR, and hemoglobin suggest that systemic metabolic abnormalities, vascular dysfunction, and anemia may contribute to impaired muscle oxygenation [42], [43], [44].

Finally, we assessed the predictive potential of SO₂ for sarcopenia diagnosis. Although the AUC was modest at 0.63, logistic regression analysis confirmed that SO₂ was an independent protective factor, with each 1 % increment associated with approximately 10 % lower risk of sarcopenia. These findings highlight that muscle hypoxia, as detected by photoacoustic imaging, is closely related to the presence of sarcopenia. Nevertheless, the modest diagnostic accuracy suggests that SO₂ should be integrated with other clinical and functional assessments to improve risk stratification and decision-making. Notably, as a noninvasive and rapid imaging modality, PAI may offer unique advantages for large-scale screening and longitudinal monitoring of sarcopenia in clinical practice.

While our study provides important insights into the role of muscle oxygenation in sarcopenia, several strengths and limitations should be acknowledged. One major strength lies in the novel application of PAI to assess skeletal muscle oxygenation in vivo, building on previous studies that have explored PAI/MSOT for muscle oxygenation in other conditions, such as peripheral artery disease, Duchenne muscular dystrophy, and healthy volunteers [24], [26], [45], [46]. However, although our sample size reached more than 179 participants, it remains modest for subgroup analyses. Moreover, our inclusion criteria were stringent, requiring all patients to have type 2 diabetes mellitus with a disease duration of at least 10 years. This specific cohort was difficult to recruit, and while clinically meaningful, this inevitably limited the sample size and may affect the generalizability of our findings. Future multicenter collaborations with larger and more diverse cohorts will be essential to validate and extend our results. Moreover, although our current findings highlight the value of PAI in phenotype analysis of sarcopenia based on muscle oxygenation, an important future direction will be to investigate whether SO₂ can objectively assess the severity of sarcopenia according to standardized criteria, such as AWGS. This will require longitudinal study designs with larger sample sizes, encompassing the full spectrum of the disease from early-stage sarcopenia to advanced stages.

## 5 Conclusion

This study demonstrates that skeletal muscle oxygenation, as measured by photoacoustic imaging, offers valuable insight into the microcirculatory and metabolic alterations associated with sarcopenia in patients with T2DM. The oxygenation phenotypes identified by clustering analysis were closely linked to clinical performance, and reduced SO₂ levels were independently associated with sarcopenia risk. These findings highlight the potential of PAI-derived SO₂ as a novel biomarker for sarcopenia assessment and pave the way for future studies to further explore its clinical utility and pathophysiological significance.

## Financial support

No.

## CRediT authorship contribution statement

**Linxin Yang:** Writing – review & editing, Writing – original draft, Investigation, Data curation, Conceptualization. **Han Wu:** Investigation, Funding acquisition. **chen jing:** Writing – review & editing, Writing – original draft, Visualization. **Fajin Dong:** Formal analysis, Data curation, Conceptualization. **Ning Lin:** Investigation, Funding acquisition, Formal analysis, Data curation, Conceptualization. **Fengyi Yuan:** Funding acquisition, Formal analysis, Data curation, Conceptualization. **Wei Wang:** Conceptualization. **Zhenxiu Zhang:** Investigation. **Jiaping Feng:** Supervision. **Wanbing Qiu:** Methodology.

## Declaration of Competing Interest

The authors declare that they have no known competing financial interests or personal relationships that could have appeared to influence the work reported in this paper.:

## Data Availability

Data will be made available on request.

## References

[1] Sayer A.A., Cooper R., Arai H. (Sep 19 2024). Sarcopenia. Nat. Rev. Dis. Prim..

[2] Sayer A.A., Cruz-Jentoft A. (Oct 6 2022). Sarcopenia definition, diagnosis and treatment: consensus is growing. Age Ageing.

[3] Izzo A., Massimino E., Riccardi G., Della Pepa G. (Jan 9 2021). A narrative review on sarcopenia in type 2 diabetes mellitus: prevalence and associated factors. Nutrients.

[4] Nishikawa H., Fukunishi S., Asai A. (Dec 2021). Sarcopenia, frailty and type 2 diabetes mellitus (Review). Mol. Med Rep..

[5] Cleasby M.E., Jamieson P.M., Atherton P.J. (May 2016). Insulin resistance and sarcopenia: mechanistic links between common co-morbidities. J. Endocrinol..

[6] Jung S.Y., Lee M.J., Lee S.Y. (Sep 2023). Analysis of the relationship between lower leg muscle mass and preservation of lower extremity in patients with diabetic foot ulcer. Int J. Low. Extrem Wounds.

[7] Abad-González Á.L., Veses S., Argente Pla M. (Jan 29 2025). Medical nutrition therapy and physical exercise for acute and chronic hyperglycemic patients with sarcopenia. Nutrients.

[8] Cheng K.Y., Chow S.K., Hung V.W. (Dec 2021). Diagnosis of sarcopenia by evaluating skeletal muscle mass by adjusted bioimpedance analysis validated with dual-energy X-ray absorptiometry. J. Cachex.. Sarcopenia Muscle.

[9] Di Vincenzo O., Marra M., Di Gregorio A., Pasanisi F., Scalfi L. (May 2021). Bioelectrical impedance analysis (BIA) -derived phase angle in sarcopenia: a systematic review. Clin. Nutr..

[10] Fu H., Wang L., Zhang W., Lu J., Yang M. (Feb 2023). Diagnostic test accuracy of ultrasound for sarcopenia diagnosis: a systematic review and meta-analysis. J. Cachex.. Sarcopenia Muscle.

[11] Ticinesi A., Meschi T., Narici M.V., Lauretani F., Maggio M. (Apr 1 2017). Muscle ultrasound and sarcopenia in older individuals: a clinical perspective. J. Am. Med Dir. Assoc..

[12] Wang S., Xu X., Cao S., Cheng J., Wang Y., Dong Y. (2024). Sonographic methods to predict type 2 diabetes patients with sarcopenia: b mode ultrasound and shear wave elastography. Clin. Hemorheol. Micro.

[13] Yi J., Shin Y., Hahn S., Lee Y.H. (Mar 4 2022). Deep learning based sarcopenia prediction from shear-wave ultrasonographic elastography and gray scale ultrasonography of rectus femoris muscle. Sci. Rep..

[14] Attia A.B.E., Balasundaram G., Moothanchery M. (Dec 2019). A review of clinical photoacoustic imaging: current and future trends. Photoacoustics.

[15] Steinberg I., Huland D.M., Vermesh O., Frostig H.E., Tummers W.S., Gambhir S.S. (Jun 2019). Photoacoustic clinical imaging. Photoacoustics.

[16] Lin L., Yao J., Li L., Wang L.V. (Jun 2016). In vivo photoacoustic tomography of myoglobin oxygen saturation. J. Biomed. Opt..

[17] Wang X., Xie Z., Zhang J., Huang Y., Dong L., Gong X. (Aug 2023). Single-wavelength sO(2) measurement based on Grüneisen-relaxation photoacoustic effect. J. Biophotonics.

[18] Gröhl J., Schellenberg M., Dreher K., Maier-Hein L. (Jun 2021). Deep learning for biomedical photoacoustic imaging: a review. Photoacoustics.

[19] Yang M., Zhao C., Wang M. (Jan 2023). Synovial oxygenation at photoacoustic imaging to assess rheumatoid arthritis disease activity. Radiology.

[20] Huang Z., Liu D., Mo S. (Aug 2024). Multimodal PA/US imaging in rheumatoid arthritis: enhanced correlation with clinical scores. Photoacoustics.

[21] Han S., Lee H., Kim C., Kim J. (Apr 22 2022). Review on multispectral photoacoustic analysis of cancer: thyroid and breast. Metabolites.

[22] Zhang M., Chen Y., Xie W., Wu S., Liao J., Cheng Q. (Jun 2021). Photoacoustic power azimuth spectrum for microvascular evaluation. Photoacoustics.

[23] Chlis N.K., Karlas A., Fasoula N.A. (Dec 2020). A sparse deep learning approach for automatic segmentation of human vasculature in multispectral optoacoustic tomography. Photoacoustics.

[24] Regensburger A.P., Fonteyne L.M., Jüngert J. (Dec 2019). Detection of collagens by multispectral optoacoustic tomography as an imaging biomarker for duchenne muscular dystrophy. Nat. Med.

[25] Regensburger A.P., Wagner A.L., Danko V. (Mar 2022). Multispectral optoacoustic tomography for non-invasive disease phenotyping in pediatric spinal muscular atrophy patients. Photoacoustics.

[26] Günther J.S., Knieling F., Träger A.P. (May 2023). Targeting muscular hemoglobin content for classification of peripheral arterial disease by noninvasive multispectral optoacoustic tomography. JACC Cardiovasc Imaging.

[27] Chen L.K., Woo J., Assantachai P. (Mar 2020). Asian working group for sarcopenia: 2019 consensus update on sarcopenia diagnosis and treatment. J. Am. Med Dir. Assoc..

[28] Coletti C., Acosta G.F., Keslacy S., Coletti D. (Feb 28 2022). Exercise-mediated reinnervation of skeletal muscle in elderly people: an update. Eur. J. Transl. Myol..

[29] Nijholt W., Scafoglieri A., Jager-Wittenaar H., Hobbelen J.S.M., van der Schans C.P. (Oct 2017). The reliability and validity of ultrasound to quantify muscles in older adults: a systematic review. J. Cachex.. Sarcopenia Muscle.

[30] Ticinesi A., Narici M.V., Lauretani F. (Dec 2018). Assessing sarcopenia with vastus lateralis muscle ultrasound: an operative protocol. Aging Clin. Exp. Res.

[31] Zhao R., Li X., Jiang Y. (2022). Evaluation of appendicular muscle mass in sarcopenia in older adults using ultrasonography: a systematic review and Meta-Analysis. Gerontology.

[32] Bellew J.W., Cayot T., Brown K. (Aug 2021). Changes in microvascular oxygenation and total hemoglobin concentration of the vastus lateralis during neuromuscular electrical stimulation (NMES). Physiother. Theory Pr..

[33] Gunton J.E. (Oct 1 2020). Hypoxia-inducible factors and diabetes. J. Clin. Invest.

[34] McGarry T., Biniecka M., Veale D.J., Fearon U. (Sep 2018). Hypoxia, oxidative stress and inflammation. Free Radic. Biol. Med.

[35] Attaway A.H., Bellar A., Mishra S. (Feb 2023). Adaptive exhaustion during prolonged intermittent hypoxia causes dysregulated skeletal muscle protein homeostasis. J. Physiol..

[36] Hong X., Isern J., Campanario S. (Sep 1 2022). Mitochondrial dynamics maintain muscle stem cell regenerative competence throughout adult life by regulating metabolism and mitophagy. Cell Stem Cell.

[37] Lowe D.A., Warren G.L., Ingalls C.P., Boorstein D.B., Armstrong R.B. (Oct 1995). Muscle function and protein metabolism after initiation of eccentric contraction-induced injury. J. Appl. Physiol. (1985).

[38] Yin L., Li N., Jia W. (Oct 2021). Skeletal muscle atrophy: from mechanisms to treatments. Pharm. Res.

[39] Park Y., Choi J.E., Hwang H.S. (Nov 1 2018). Protein supplementation improves muscle mass and physical performance in undernourished prefrail and frail elderly subjects: a randomized, double-blind, placebo-controlled trial. Am. J. Clin. Nutr..

[40] Wandrag L., Siervo M., Riley H.L. (Oct 2017). Does hypoxia play a role in the development of sarcopenia in humans? Mechanistic insights from the caudwell xtreme everest expedition. Redox Biol..

[41] Chaillou T. (2018). Skeletal muscle fiber type in hypoxia: adaptation to High-Altitude exposure and under conditions of pathological hypoxia. Front Physiol..

[42] Picca A., Coelho-Junior H.J., Calvani R., Marzetti E., Vetrano D.L. (Jan 2022). Biomarkers shared by frailty and sarcopenia in older adults: a systematic review and meta-analysis. Ageing Res Rev..

[43] Yang R., Zhang Y., Shen X., Yan S. (Aug 2016). Sarcopenia associated with renal function in the patients with type 2 diabetes. Diabetes Res Clin. Pr..

[44] Malone J.I., Hansen B.C. (Feb 2019). Does obesity cause type 2 diabetes mellitus (T2DM)? or is it the opposite?. Pedia Diabetes.

[45] Karlas A., Fasoula N.A., Katsouli N. (Apr 2023). Skeletal muscle optoacoustics reveals patterns of circulatory function and oxygen metabolism during exercise. Photoacoustics.

[46] Helfen A., Masthoff M., Claussen J. (Jan 9 2019). Multispectral optoacoustic tomography: Intra- and interobserver variability using a clinical hybrid approach. J. Clin. Med.

