# Supplementary material for: Muscle oxygen saturation stratification by photoacoustic imaging in diabetic sarcopenia: Association with disease status
Source: Photoacoustics. 2025 Sep 27;46:100771. doi: 10.1016/j.pacs.2025.100771 (PMC12514347; doi:10.1016/j.pacs.2025.100771)
Supplement: Supplementary file 1 — Supplementary material [file mmc1.pdf]

Appendix E1:

Imaging Settings and Oxygen Saturation (SO_2) Measurement for Muscle Oxygenation

Imaging System and Setup

The system has horizontal and vertical resolutions of less than 1 mm, and a signal-to-noise ratio of 27.5 dB within the depth range from 5 mm to 20 mm. The imaging parameters were set to optimize the photoacoustic signal from muscle tissue. The imaging gain was adjusted from 55 to 45 dB to reduce background noise, particularly under the bone surfaces.

Dual-Wavelength PAI for SO_2 Measurement

Photoacoustic imaging was performed using dual-wavelength excitation at 750 nm and 830 nm to quantify tissue oxygen saturation (SO_2). The system provided real-time imaging of muscle tissue, with acquired images displayed as pseudo-color maps superimposed on grayscale ultrasound images. Red and blue colors on the images represented high and low SO_2 levels, respectively, visually indicating the muscle oxygenation status.

Oxygen Saturation (SO_2) Calculation

Oxygen saturation (SO_2) was calculated using the following formula based on the light absorption properties of oxyhemoglobin (HbO_2) and deoxyhemoglobin (Hb) at the two wavelengths:

$$SO_2 = HbO_2 / (HbO_2 + Hb) \times 100\%$$

The light absorption of oxyhemoglobin (HbO_2) and deoxyhemoglobin (Hb) varies markedly at the wavelength of 600–700 nm and become identical in the infrared range. In this system, the SO_2 values were calculated using the signals of 750 nm (λ1) and 830 nm (λ2), expressed as the following equation:

$$SO_2 = \frac{C_{HbO2}}{C_{Hb} + C_{HbO2}} = \frac{\varepsilon_{Hb}^{\lambda_1} A^{\lambda_2} - \varepsilon_{Hb}^{\lambda_2} A^{\lambda_1}}{A^{\lambda_1}\left(\varepsilon_{HbO2}^{\lambda_2} - \varepsilon_{Hb}^{\lambda_2}\right) + A^{\lambda_2}\left(\varepsilon_{Hb}^{\lambda_1} - \varepsilon_{HbO2}^{\lambda_1}\right)} \times 100\%$$

(CHb and CHbO2: Hb and HbO2 content; $\varepsilon_{Hb}^{\lambda1}$, $\varepsilon_{Hb}^{\lambda2}$, $\varepsilon_{HbO2}^{\lambda1}$, $\varepsilon_{HbO2}^{\lambda2}$: extinction coefficients of Hb and HbO2 at λ1 and λ2; Aλ1and Aλ2: photoacoustic intensity at wavelengths λ1 and λ2).

The SO_2 values were automatically computed by an integrated algorithm based on the photoacoustic signals at 750 nm and 830 nm wavelengths. The accuracy of the measurements was validated in previous experiments using phantoms and animal models.

Image Acquisition and Muscle Oxygenation Assessment

The photoacoustic imaging system was used to assess the muscle oxygenation of the vastus lateralis and gastrocnemius muscles. The imaging was performed at four predefined anatomical sites for each patient: transverse and longitudinal planes of both the vastus lateralis and gastrocnemius muscles. For each region of interest (ROI), three separate measurements of SO_2 were taken by the ultrasound physicians. The average SO_2 value was then calculated for each ROI. The overall muscle oxygenation for each patient was expressed as the mean SO_2 across the four measurement sites.

Unsupervised Clustering of SO_2 Values

To investigate oxygenation patterns, an unsupervised K-means clustering analysis was applied to the SO_2 values obtained from sarcopenic patients. The optimal number of clusters, determined using the elbow method and clustering performance indices, was found to be three. These clusters corresponded with the following oxygenation phenotypes:

- Hypoxia (low oxygenation): Indicated by blue signals on pseudo-color images.
- Intermediate Oxygenation: Indicated by mixed colors on pseudo-color images.
- Hyperoxia (high oxygenation): Indicated by red signals on pseudo-color images.

Reliability of SO_2 Measurement

Two experienced sonographers measured the SO_2 value of each region of interest (ROI) separately, and the intraclass correlation coefficient (ICC) of SO_2 measurements was 0.93, indicating good reliability of repeated measurements.
